# Trends in the process and outcome indicators of type 2 diabetes care: a cohort study from Eastern Finland, 2012–2017

**DOI:** 10.1186/s12875-020-01324-5

**Published:** 2020-12-04

**Authors:** Marja-Leena Lamidi, Katja Wikström, Laura Inglin, Päivi Rautiainen, Hilkka Tirkkonen, Tiina Laatikainen

**Affiliations:** 1 Institute of Public Health and Clinical Nutrition, University of Eastern Finland, PO Box 1627, FI-70211 Kuopio, Finland; 2 Department of Public Health Solutions, Finnish Institute for Health and Welfare, PO Box 30, FI-00271 Helsinki, Finland; 3 Joint Municipal Authority for North Karelia Social and Health Services, Tikkamäentie 16, FI-80210 Joensuu, Finland

**Keywords:** Type 2 diabetes mellitus, Care, HbA1c, LDL, Trends, Electronic health records

## Abstract

**Background:**

Evidence-based guidelines include concrete treatment targets that can be used as process and outcome indicators in the evaluation of the quality of healthcare services and diabetes care. Quality improvement can be evaluated by monitoring longitudinal trends in the care indicators on the system level. The aim of this study is to describe trends in the processes and outcomes of care among people with type 2 diabetes in North Karelia, Finland.

**Methods:**

The data consist of all adults with type 2 diabetes (identified from the EHRs using ICD-10 codes) who used primary or specialized care services in North Karelia during 2012–2017. The diabetes care was evaluated using the measurement activity, treatment levels, and the achievement of the treatment targets for HbA1c and LDL as care indicators. Logistic and linear models with generalized estimating equations were used to assess the differences between years, sexes, and age groups.

**Results:**

The proportion of patients with annual measurement varied between 75.8 and 78.1% for HbA1c and between 67.4 and 69.1% for LDL during a five-year follow-up. The changes in average levels were moderate: a 0.2% (2 mmol/mol) increase for HbA1c and a 0.1 mmol/l decrease for LDL. Anyway, the proportion of patients meeting the treatment target for HbA1c decreased from 72.7 to 67.3% (age-adjusted decrease: 5.7%p, 95% CI: 4.5–6.9) and for LDL it increased from 53.4 to 59.5% (age-adjusted increase: 5.6%p, 95% CI: 4.2–7.0). Women were measured and met the HbA1c target level more often compared with men. Conversely, men met the LDL target level more often than women, and the age-adjusted difference between sexes increased smoothly from 7.9%p to 11.7%p.

**Conclusions:**

The achievements in relation to type 2 diabetes care in North Karelia are very good, but no major improvement was observed during follow-up. HbA1c levels had a rising tendency and LDL levels declining tendency indicating quality improvement in LDL management, but challenges in further improvement in glucose control.

## Background

Type 2 diabetes is a major public health problem both in Finland and worldwide, which strains healthcare systems. It is a progressive disease that is characterized by the development of severe and expensive complications, such as strokes and cardiovascular disease [1, 2]. Complications associated with type 2 diabetes can be prevented, or at least postponed, by the good care of diabetes [3]. The good care of type 2 diabetes includes several components, such as the maintenance of good glycemic, lipid, and blood pressure control by self-management and by regular monitoring in healthcare [4]. Evidence-based guidelines have been published internationally and nationally to support the treatment and care decision-making among healthcare professionals [4–7]. According to the guidelines of American Diabetes Association [6] and Finnish Current Care Guidelines [5], lifestyle management, including changes in diet and physical activity, weight management and smoking cessation, combined with suitable pharmacological treatment are the cornerstones of diabetes care.

Guidelines include a set of process and outcome indicators that are intended as a basis for good diabetes care and are also useful measures in the evaluation of the quality of healthcare services and diabetes care. According to the Finnish Current Care Guidelines for diabetes [5], the glycated hemoglobin (HbA1c) level should be lower than 7.0% (53 mmol/mol), measured with a measurement frequency of once or twice a year. The recommended level for low-density lipoproteins (LDLs) is ≤2.5 mmol/l and it should be measured at least once in one to 3 years. However, the treatment goals are supposed to be tailored for each patient based on his or her risk-factor levels; the risk factors include age, life expectancy, the duration of the disease, the severity of the disease and comorbidities, and for example LDL should be lower than 1.8 mmol/l if type 2 diabetes patient has also increased cardiovascular risk or CVD, as well the target of blood pressure is set by the patient’s CVD risk. The new international guidelines set the target for LDL even lower than the Finnish Current Care Guidelines [8]. However, the existence of clinical guidelines does not alone guarantee quality of care. In fact, they have been shown to improve clinical practice only when introduced in the context of evaluation.

In order to evaluate the quality of diabetes care, several sets of indicators have been developed and incorporated into the national and international guidelines. In the United States, the Diabetes Quality Indicator Project developed and implemented a comprehensive set of process and outcome indicators for care evaluation [9]. The OECD Quality Indicator Project revised this set and published a list of nine health system level quality indicators for diabetes care [10]. Process indicators consisted of annual testing of HbA1c and LDL, screening for nephropathy, and eye examination. The control of HbA1c and LDL were defined as outcome indicators. In addition, the OECD diabetes panel proposed the comorbidities of diabetes, such as lower extremity amputation and kidney disease, and CVD mortality to be used as new long-term indicators for diabetes care. These health system level quality indicators have been accepted internationally.

Monitoring longitudinal trends in the processes and treatment outcomes of diabetes care is possible by using follow-up data. Further, follow-up data can indicate areas in need of improvement and enable the evaluation of improvement work. However, not all countries have such data available. There are countries with national diabetes registers, like Norway [11], Denmark [12], Sweden [13], Australia [14], England and Wales [15], and the USA [16]. However, the coverage and content of these registers varies. Not all of them include indicators reflecting the achievement of medical goals, and therefore, they do not all allow the long-term evaluation of the treatment of diabetes and its associated conditions.

In Finland, there is no diabetes register, and previous studies on the care processes and outcomes are scarce and based on separate evaluations [17], although the amount of people with type 2 diabetes has been increasing during recent decades. The rising prevalence can be explained by several factors, such as increased awareness and new practices leading to more testing and detection of new cases, increased life expectancy of people with diabetes and comorbidities, and a real increase in new cases due to the ageing of the population and urbanization, which causes changes in environment and lifestyles [18]. Despite these changes in the population over time, the main goal of the care systems is to provide a high quality of care for all patients with diabetes with the existing resources.

In the region of the Joint Municipal Authority for North Karelia Social and Health Services in Finland, the electronic health records (EHRs) has covered all type 2 diabetes patients living in the area and included data from both primary and specialized care since 2011. This regional database of EHRs has made follow-up analyses possible from health system perspective. The aim of this study was to evaluate the care processes and outcomes by describing the trends in the measurement activity, treatment levels, and the achievement of the treatment targets for both HbA1c and LDL among all type 2 diabetes patients in the region of North Karelia utilizing EHR data from 2012 to 2017.

## Research design and methods

### The data source and study population

In Finland, municipalities are responsible for organizing and financing health care and most of the health services are public providing residents an equal access to services. Type 2 diabetes patients are treated mainly in primary health care by a team of professionals and no incentives are in use. North Karelia is a region in eastern Finland consisting of 14 municipalities. In North Karelia, the regional database was established in the beginning of 2011 and includes data on patient’s age, sex, place of residence, permanent diagnoses, laboratory markers and prescriptions.

All adult patients with type 2 diabetes in 2012 were identified from the regional EHRs using the ICD-10 code E11. These patients were followed until 2018 and in each year newly diagnosed patients were included in the data and those who had died were excluded from the data. Every year those who were alive at the end of the year and who had been diagnosed before the beginning of the year were included in the analyses, that is, patients with whole-year follow-up.

Those who were not living permanently in the study region were excluded. Information on the municipality of domicile was retrieved thrice: in the years 2013, 2016, and 2018. The last-collected municipality information was used for the years 2016 and 2017 and for those who had died before 2018. The municipality information from 2016 was used for 2015 and in 2013–2014 for those who were diagnosed after 2012, and information from 2013 was used for remaining years 2012–2014.

### Variables

From the EHRs, patients’ age, sex, and the process and outcome indicators for type 2 diabetes care were obtained. The annual measurement activity of HbA1c and LDL (the proportion of patients measured) were used as process indicators, and the treatment outcomes of care were assessed by the mean levels of HbA1c and LDL, and by the achievement of the target levels for HbA1c and LDL (HbA1c < 7% [53 mmol/mol]; LDL < 2.5 mmol/l) during the follow-up period. The used process and outcome indicators originated from the OECD’s health system level quality indicators [10] and were defined based on the recommendations of the Finnish Current Care Guidelines for Diabetes [5]. For each year, the last LDL and HbA1c measurements were included in the analyses for each patient so that patients that were measured more often were not overrepresented in data. Only HbA1c measurements that were taken at least 3 months after the diagnosis of diabetes and LDL measurements that were taken at least 1 month after diagnosis were considered. Annual statin treatments were defined using the prescriptions and identified with ATC codes to see the trend in the use of statins during the follow-up period. All laboratory samples were analyzed in the Eastern Finland Laboratory Centre Joint Authority Enterprise (ISLAB), which is an accredited laboratory that participates in external quality surveys, and the results of laboratory analyses were entered straight from the laboratory to the EHR’s.

### Statistical analyses

Counts and percentages were used to describe binary data, such as sex, process indicators (whether measured or not), and outcome indicators (whether the target has been met or not). Percentage points (%p) were used for the differences between percentages. As continuous variables, HbA1c and LDL both had skewed distributions. The logarithmic transformations (lg10(LDL) and lg10(HbA1c-20) for the unit (mmol/mol)) were used to make them more symmetric. Means were used for these transformations and back transformations were used to get them back to the original scales (the geometric mean). To see the effect of age, age was categorized in 10 years intervals: under 40, 40–49, 50–59, 60–69, 70–79, 80–89, and 90 years old or older.

A logistic model with generalized estimating equations (GEEs) was used for the binary variables (sex, whether measured or not, whether or not the target has been met) and a linear model with GEEs was used for the continuous variables (age and logarithmic of LDL and HbA1c) in order to capture the changes between the years, sexes, and age groups. GEEs can model data where most of the patients have more than one observation. The R language and environment for statistical computing (Version 3.5.3) [19] and IBM SPSS Statistics for Windows (Version 25.0) [20] were used in statistical analyses. *P*-values of 0.05 or less were regarded as statistically significant and 95% confidence intervals (CI) were used for statistics.

## Results

### Population characteristics

The number of patients with type 2 diabetes in North Karelia increased from 11,299 to 12,800 during the follow-up years (2012–2017; see Table 1). There were less women and the proportion of them dropped smoothly by altogether 1.5%p in 5 years (from 47.1 to 45.6%). Mean age increased by 1.2 years, from 67.8 to 69.0 years old. The women were older than the men, although the mean age difference between sexes decreased from 4.2 years to 3.5 years during the follow-up (*p* < 0.001 for sex*year interaction). So, the increase in mean age was 1.6 years for men and 0.8 years for women.

**Table 1:** Population description, and statistics for the HbA1c and LDL measurements for patients with type 2 diabetes in North Karelia, Finland during 2012 to 2017

		2012	2013	2014	2015	2016	2017	***P***-value
Number of all patients	*N*	11,806	12,794	13,141	13,221	13,943	14,116	
Diagnosed in this year		577	985	860	753	783	751	
Died in this year			514	513	510	579	574	
Diagnosed or dead			1483	1360	1254	1353	1316	
**Patients in the whole-year follow-up**		**11,229**	**11,311**	**11,781**	**11,967**	**12,590**	**12,800**	
**Sex**	Women, %	47.1	46.9	46.5	46.0	45.8	45.6	< 0.001
**Age, years**	Mean	67.8	68.1	68.3	68.6	68.7	69.0	< 0.001
Women		70.0	70.2	70.5	70.6	70.7	70.9	< 0.001
Men		65.8	66.3	66.5	66.9	67.0	67.4	< 0.001
**HbA1c, % (mmol/mol)**	Measured, %	78.1	76.2	76.1	76.8	75.8	76.1	< 0.001
	Mean	6.64	6.62	6.74	6.75	6.72	6.80	
		(49.0)	(48.8)	(50.1)	(50.3)	(49.9)	(50.8)	
	Geom. mean	6.42	6.40	6.54	6.54	6.50	6.59	
		(46.7)	(46.4)	(47.9)	(48.0)	(47.5)	(48.5)	< 0.001
	< 7 (53), %	72.7	72.4	69.7	68.5	69.7	67.3	< 0.001
Women	Measured, %	80.1	78.6	78.6	79.6	77.0	77.8	< 0.001
	Mean	6.60	6.60	6.73	6.73	6.69	6.78	
		(48.6)	(48.6)	(50.0)	(50.0)	(49.6)	(50.6)	
	Geom. mean	6.39	6.38	6.53	6.52	6.48	6.57	
		(46.3)	(46.2)	(47.9)	(47.7)	(47.3)	(48.3)	< 0.001
	< 7 (53), %	74.6	73.3	70.5	70.4	71.1	69.2	< 0.001
Men	Measured, %	76.2	74.2	74.0	74.5	74.7	74.8	0.012
	Mean	6.68	6.64	6.75	6.78	6.74	6.82	
		(49.5)	(49.1)	(50.3)	(50.5)	(50.1)	(51.0)	
	Geom. mean	6.45	6.41	6.54	6.56	6.52	6.61	
		(47.0)	(46.6)	(48.0)	(48.2)	(47.7)	(48.7)	< 0.001
	< 7 (53), %	70.9	71.5	69.0	66.8	68.4	65.6	< 0.001
**LDL, mmol/l**	Measured, %	69.1	67.5	67.4	67.6	67.4	68.5	0.003
	Mean	2.52	2.50	2.50	2.45	2.45	2.42	
	Geom. mean	2.38	2.36	2.36	2.31	2.30	2.27	< 0.001
	< 2.5, %	53.4	55.0	55.1	57.4	57.5	59.5	< 0.001
Women	Measured, %	69.0	67.4	66.9	68.2	67.0	68.9	0.012
	Mean	2.61	2.58	2.59	2.57	2.56	2.53	
	Geom. mean	2.47	2.44	2.44	2.42	2.41	2.38	< 0.001
	< 2.5, %	50.3	51.6	51.4	52.6	52.8	54.1	0.010
Men	Measured, %	69.2	67.5	67.8	67.0	67.8	68.1	0.095
	Mean	2.44	2.43	2.42	2.35	2.36	2.32	
	Geom. mean	2.31	2.30	2.28	2.22	2.22	2.18	< 0.001
	< 2.5, %	56.1	58.0	58.4	61.7	61.4	64.1	< 0.001

### The process and outcome indicators for type 2 diabetes care over time

**Fig. 1:**
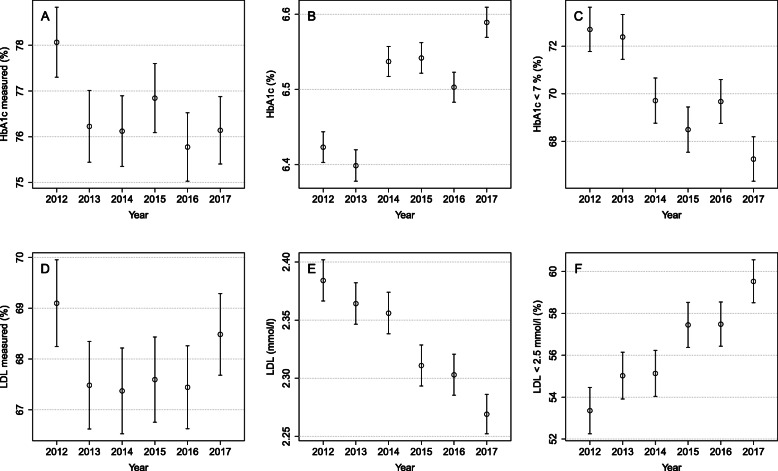
The proportions of measured (**a** and **d**), geometric means (**b** and **e**), and the proportions that meet the target (**c** and **f**) with a 95% CI for HbA1c and LDL (correspondingly) during 2012 to 2017

Annual measurement activity was quite stable during the years 2012–2017; it was highest in the year 2012 for both HbA1c and LDL (see Table 1). The proportion of those whose HbA1c was measured varied between 75.8 and 78.1% (see Fig. 1a). LDL was measured less frequently, fluctuating over the years from 67.4 to 69.1% (see Fig. 1d).

HbA1c levels fluctuated with an increasing tendency while LDL decreased steadily (*p* < 0.001 for the linear trend for both) (see Fig. 1b, e). The overall changes in average levels were quite moderate: a 0.2% (2 mmol/mol) increase for HbA1c and a 0.1 mmol/l decrease for LDL. Clearer changes were seen in the proportions of patients who achieved the treatment targets over time. The proportion of patients who achieved the target HbA1c level of less than 7% (53 mmol/mol), reduced by 5.4%p (95% CI: 4.3–6.6) from 72.7 to 67.3% in 5 years (see Fig. 1c). Correspondingly, the proportion of patients who achieved the target in LDL level of less than 2.5 mmol/l increased by 6.1%p (95% CI: 4.8–7.5), from 53.4 to 59.5% (see Fig. 1f).

### Process and outcome indicators over time among women and men

**Fig. 2:**
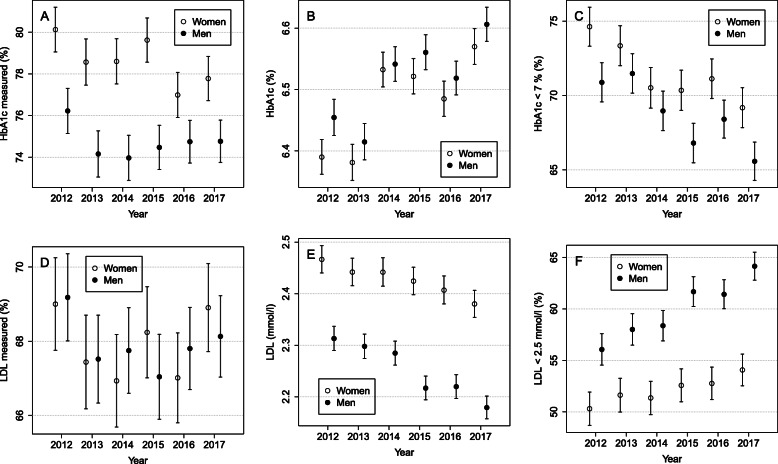
The proportions of measured (**a** and **d**), geometric means (**b** and **e**), and the proportions that meet the target (**c** and **f**) with a 95% CI for HbA1c and LDL (correspondingly) by sex during 2012 to 2017

HbA1c was more often measured among women than among men (see Fig. 2a): the biggest difference in the measurement activity between sexes was 5.1%p in the year 2015. LDL measurement activities were not statistically significantly different between sexes (see Fig. 2d). Women more frequently achieved the target level of HbA1c being less than 7% (53 mmol/mol); the overall difference between sexes was 2.7%p (95% CI: 1.5–4.2) over the years (see Fig. 2c). On the other hand, men achieved the target level of LDL (less than 2.5 mmol/l) more frequently compared with women and the difference between sexes increased over the years from 5.7%p to 10.1%p (*p* < 0.001 for sex*year interaction) (see Fig. 2f). Thus, the increase in the proportion of patients who achieved the target level in LDL was more than twofold in men compared with women: 8.1%p (95% CI: 6.2–9.9) vs. 3.8%p (95% CI: 1.8–5.8) during the 5 years.

The amount of statin prescriptions was analyzed separately to see the overall trend in the use of statins in North Karelia. The amount of statin prescriptions for type 2 diabetes patients has increased, especially in the two first years of this study period and increasing altogether by about 5.3%p during the follow-up period, from 65.7 to 71.0%. The difference between men and women was about 5%p and remained quite stable over the years.

### Process and outcome indicators by age group among women and men

**Fig. 3:**
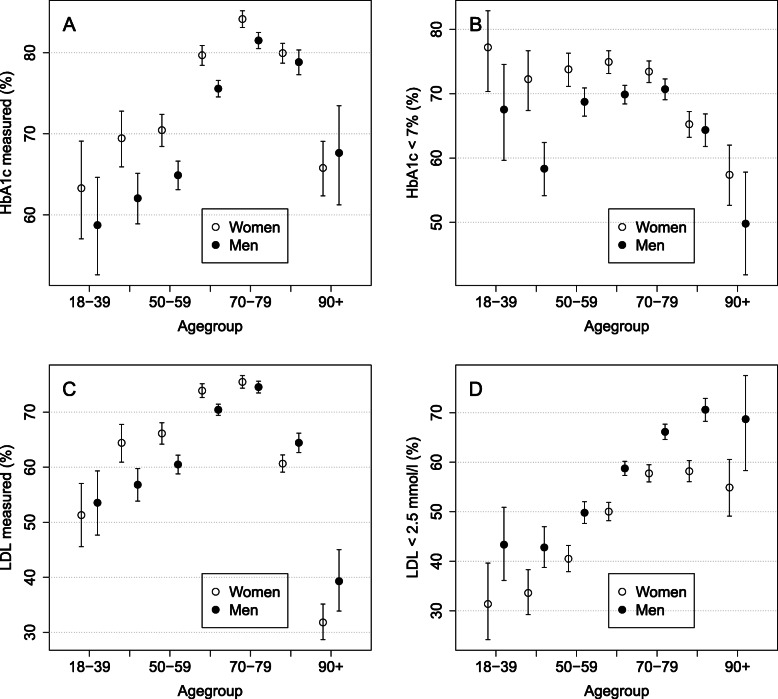
The proportions of measured (**a** and **c**) and the proportions that meet the target (**b** and **d**) with a 95% CI for HbA1c and LDL (correspondingly) by age group

Overall, for the whole time period from 2012 to 2017, age was related to measurement activity and treatment levels (see Fig. 3). When looking at age groups in 10 years intervals (< 40, 40–49, 50–59, 60–69, 70–79, 80–89, 90+), the proportion of patients with annual measurement of HbA1c was lowest in the youngest category (63% for women and 59% for men), increasing quite smoothly up to the age group 70–79 (84% for women and 82% for men) and decreasing after that. A similar pattern was observed for annual measures of LDL: it reached a peak in the age group 70–79 (76% for women and 75% for men) but had the lowest proportion in the oldest age group (32% for women and 39% for men).

The proportion of those who achieved the target level of less than 7% (53 mmol/mol) HbA1c was the lowest in the oldest age group (57% for women and 50% for men) and the highest in the youngest age group among women (77%) and in the age group 70–79 among men (71%). The proportion of patients who achieved the target level of less than 2.5 mmol/l in LDL also increased by age; the proportion was lowest in the youngest age group (31% for women and 43% for men) and the highest in the second to last age group (58% for women and 71% for men).

### Age-adjusted changes in process and outcome indicators

The overall age-adjusted change in the proportion of patients who achieved the treatment target was not much different from the non-adjusted results: 5.7%p (95% CI: 4.5–6.9) decrease for HbA1c and 5.6%p (95% CI: 4.2–7.0) increase for LDL. In contrast to the overall picture, the difference between sexes became more obvious after age adjustments. The age-adjusted difference between women and men in HbA1c measuring frequency was slightly higher than non-adjusted: 5.6%p (95% CI: 3.8–7.3) in the year 2015, when the difference between sexes was biggest. The difference in LDL measuring frequency became statistically significant (*p* < 0.001); the overall age-adjusted change was 2.0%p (95% CI: 0.9–3.1) bigger among women compared with men. Also, the sex difference in the treatment outcome indicators increased. The overall age-adjusted difference between sexes (women vs. men) in the proportion of patients who achieved the target of HbA1c level was 4.2%p (95% CI: 2.8–5.7). The age-adjusted difference between sexes (men vs. women) in the proportion of patients who met the LDL target increased from 7.9%p to 11.7%p. Age-adjusted decreases of LDL levels from 2012 to 2017 were a bit smaller: 3.4%p (95% CI: 1.4–5.4) for women and 7.2%p (95% CI: 5.3–9.1) for men.

## Discussion

In this EHR-based study, the processes and outcomes of care were evaluated among all type 2 diabetic patients who were living in the North Karelia during the five-year follow-up period. The measurement activity, treatment levels, and the achievement of the treatment targets for HbA1c and LDL were used as measures of good care of diabetes, and the selected measures originated from the internationally accepted health system level quality indicators of care [10].

North Karelia is one of the first areas in Finland to have prepared for the impending national social and health care reform, aiming at the integration of social and health services by establishing an integrated EHR system. The EHR covered the whole region at the beginning of the year 2011. The data from EHRs were used to identify differences in the detection of type 2 diabetes and evidence treatment gaps. There were substantial regional differences in the identification of type 2 diabetes in the region of North Karelia in 2012, but the detection of type 2 diabetes improved and the regional differences declined due to quality improvement activities, such as guidance given by the diabetologist in charge in order to improve detection practices [21]. As a result, the amount of new diabetes cases was highest in 2013 and leveled off after that at a higher level than in 2012.

This study shows that the measurement activity of HbA1c and LDL was quite stable over time in the region of North Karelia, but the highest activity for both measures was seen in the year 2012. In addition to regional recommendations on the follow-up of patients, developed as part of the quality improvement activities, the new integrated EHR system, where recordings from both primary care and specialized care can be reviewed at all levels of the service system, might have (to some extent) reduced the frequency of laboratory assessments among patients with continuously good control of type 2 diabetes.

Previous studies have shown that the burden and complications of diabetes differ between sexes [22]. These differences could be partly explained by the differences in type 2 diabetes risk factor assessments and management. Previous studies have reported mixed findings regarding the differences in the screening activity for HbA1c or LDL between sexes among people with diabetes. In this study, HbA1c was more often measured among women than among men. After age adjustment, there was also a small difference in LDL measurement activity between sexes. Similarly, a study using healthcare effectiveness data and an information set, Medicare enrollment files, and the US Census found that women received more HbA1c screenings compared with men and cholesterol screening was similar between sexes [23]. On the other hand, the Dutch study using routinely collected data from 53 primary care practices did not find any sex differences in the assessment of HbA1c and LDL among people with type 2 diabetes [24]. The quality of diabetes care has shown to be associated with the age of patients [25]. In this study, the measurement activity of HbA1c and LDL increased with increasing age up to the age group 70–79. The increasing multimorbidity by age obviously increases the healthcare visits and thus there is more active follow-up of risk-factor levels [26, 27].

The results of this study indicate a rising tendency in HbA1c levels during follow-up and a declining tendency in LDL levels during follow-up. The overall changes in the average levels of HbA1c and LDL were quite moderate, but clear changes were seen in the proportion of patients who achieved the treatment targets over time. The slightly decreasing proportion of newly diagnosed patients during the follow-up period might partly explain the finding related to HbA1c targets as the treatment targets are usually achieved more easily among them. Our results are in agreement with previous findings showing that women achieve the HbA1c target level more often compared with men [28, 29]. Instead, men met the LDL target level more often than women, which is in line with earlier studies showing worse management of cardiovascular risk factors among women [30, 31]. Also, the recent study of Jong et al. showed that women with diabetes were less likely to achieve treatment and control for LDL, but no sex differences were found for the control of HbA1c [24]. In general, the proportion of those achieving the HbA1c target in the region of North Karelia is very high compared with many international assessments [15, 32, 33].

The study of Stone et al. analyzed the levels of adherence to recommendations for the management of type 2 diabetes in eight European countries: Belgium, France, Germany, Italy, Ireland, Sweden, the Netherlands, and the United Kingdom [32]. In these countries, the achievements of treatment targets were low, with approximately half of the total sample having both HbA1c (< 7%; 53 mmol/mol) and LDL (< 2.6 mmol/l) within target. According to the National Diabetes Audit in England and Wales, the percentage of people achieving the target of HbA1c (≤58 mmol/l) varied between 64.9 and 66.8% during 2010–2016 [15]. During the same period, the percentage of people achieving the target of LDL (< 5 mmol/l) varied between 76.7 and 78.0% [15]. The study of Mata-Cases et al. found no relevant differences in the control of HbA1c over time in Catalonia, Spain. Mean HbA1c value was around 7.2%, and the percentage of patients reaching an HbA1c ≤7% target ranged between 52.2 and 55.6% from 2003 to 2008 [34]. The study of Lipska et al. found that the overall proportion of patients with HbA1c < 7% declined from 56.4 to 54.2% during 8-year follow-up in the U.S. [35].

Factors that might explain the good control of HbA1c in North Karelia include the proper implementation of evidence-based guidelines and a multidisciplinary team approach in patient-centered care, redesigning care processes, clinical pathways and consultation practices, developing and implementing EHRs for evaluation, trainings for personnel and patient education, and public health policy that support the prevention and care of chronic diseases. Nowadays, as the targeted level varies according to patients’ age and other conditions, in accord with the recent guidelines, it might be that the room for improvement in HbA1c control starts to be very narrow [5, 6, 36].

Age increases the risk of type 2 diabetes, which has been explained by the age-related decline in beta-cell function, by an increase in the level of other risk factors, and by accumulating risk exposure during a lifetime [37, 38]. Also, the increased life expectancy of people with diabetes can partly explain the rising prevalence of type 2 diabetes [39]. In this study, the mean age of the study population increased by 1.6 years for men and 0.8 years for women during the follow-up. A similar pattern of ageing can be seen in the whole population of Finland: life expectancy has increased by 1.2 years in men and 0.8 years in women during this time period (from 2012 to 2017) [40]. Anyway, in this study ageing does not explain changes in achieving the target levels for HbA1c and LDL. After adjusting for age, about a yearly 1.1%p decrease can be seen in those achieving the target level of HbA1c. The same amount of increase can be seen in those achieving the target level for LDL, and the increase is twofold in men compared with women.

In the treatment of type 2 diabetic patients, one of the important goals is to reach a good HbA1c level. However, taking into account that with the increasing duration of disease it becomes harder to achieve and keep a good HbA1c level, reaching a good LDL level can be even more important for preventing or at least postponing complications. In the recommendations of the Finnish Current Care Guidelines for Diabetes [5] and in clinical practice, more attention is paid to not only lowering the blood glucose levels but also reducing the macrovascular risk factors, such as high lipid values and high blood pressure. Men are at higher risk of cardiovascular diseases compared with women and thus the threshold for actively treating their high LDL levels may be lower, even though the relative risks of cardiovascular diseases are considerably greater in women with diabetes than in men with diabetes [41]. In North Karelia, the amount of statin prescriptions for type 2 diabetes patients increased more among men than women during the follow-up period, and this might at least partly explain the lower levels of LDL values for men. In general, the achievements of LDL targets were moderately good and our previous study showed that 59% of women and 66% of men achieved the recommended level of LDL (≤2.5 mmol/l) in 2015–2016 [42]. In addition, a subgroup analysis of type 2 diabetes patients with CVD indicated that 22% of women and 32% of men achieved the LDL target < 1.8 mmol/l.

All Finnish residents are eligible for state reimbursement for medicine expenses related to diabetes, based upon a medical diagnosis. In 2017, the Finnish government decided to limit drug reimbursement, and therefore, the state’s share of drug expenses for the treatment of type 2 diabetes dropped from 100 to 65%. Type 2 diabetes is most common among older people, and they might have limited financial possibilities to buy medication (especially the new, more expensive medications) with limited reimbursement rights. A study by Suviranta et al. examined the effects of this reform as experienced by patients with type 2 diabetes. Almost half of the participants reported an effect, such as increased expenditure or difficulty in purchasing medicines, caused by the reimbursement reform [43]. This could be one explanation for the slightly increasing levels of HbA1c in the year 2017. Also, a major change in the organization of health services occurred when the Joint Municipal Authority for North Karelia Social and Health Services was established, joining the health service organizations of 14 municipalities in the beginning of 2017. The reorganization phase might also have had some effect, especially on the continuity of the care of patients.

### Strengths and limitations

In North Karelia, the regional database was implemented in 2009, and it has covered all of the municipalities in North Karelia from the beginning of the year 2011. The strength of this study is that the database cover all type 2 diabetes patients living in the area and includes data from both primary and specialized care, and therefore selection bias, non-responsiveness of the patients and missing laboratory data were avoided. However, the quality of manually entered information in EHRs is not yet optimal and we were not able to use information on blood pressure levels, smoking or obesity in our analysis. Also the follow-up was too short to take into account complications as treatment outcomes. Further, the study region is located in the Eastern Finland and it is not representative for the whole country. It would be important to make similar analysis nationwide, but so far such data does not exist from other regions.

## Conclusion

This study describes how the treatment processes and outcomes of type 2 diabetes care developed over time among all diagnosed type 2 diabetes patients in North Karelia. The achievements in glucose control in North Karelia were very good, but no major improvement was observed during recent years. In this study, women achieved the HbA1c target level more often compared with men, but generally, the proportion of those achieving the HbA1c target was very high in the study region compared with many international studies. Type 2 diabetes is progressive disease and maintaining appropriate HbA1c control in long-term is challenging. It might be that the room for improvement measured by HbA1c using a single cut-off level starts to be very narrow and the targeted level also varies according to patients’ age, comorbidities and other factors affecting care decisions. The improvement in LDL control was observed among men, and an increasing amount of statin prescriptions among them might at least partly explain the differences observed in LDL control between sexes. This indicates disparities in the management of diabetes among type 2 diabetes patients, and more attention should be paid to treatment of women, with declining tendency in LDL management. The information from the following years will be of great interest as in this study the follow-up period lasted to the end of the year 2017, during which time some remarkable changes happened in the service system as well as in the drug reimbursement system. This study indicates that EHRs are a valuable data source to be used to identify the trends and the differences in the processes and outcomes of diabetes care, and to guide the quality improvement work in health care systems.

## Data Availability

The health records data analyzed in the current study is confidential and according to the Personal Data act cannot be put publicly available to protect the privacy of the patients.

## Abbreviations

EHR: Electronic health records
HbA1c: Glycated hemoglobin
LDL: Low-density lipoproteins
%p: Percentage points
CI: Confidence interval
ISLAB: Eastern Finland Laboratory Centre Joint Authority Enterprise
GEEs: Generalized estimating equations
